# Analysis of a Web-Based Dashboard to Support the Use of National Audit Data in Quality Improvement: Realist Evaluation

**DOI:** 10.2196/28854

**Published:** 2021-11-23

**Authors:** Natasha Alvarado, Lynn McVey, Mai Elshehaly, Joanne Greenhalgh, Dawn Dowding, Roy Ruddle, Chris P Gale, Mamas Mamas, Patrick Doherty, Robert West, Richard Feltbower, Rebecca Randell

**Affiliations:** 1 Faculty of Health Studies University of Bradford Bradford United Kingdom; 2 Wolfson Centre for Applied Health Research Bradford United Kingdom; 3 Faculty of Engineering and Informatics University of Bradford Bradford United Kingdom; 4 School of Sociology and Social Policy University of Leeds Leeds United Kingdom; 5 School of Health Sciences University of Manchester Manchester United Kingdom; 6 School of Computing University of Leeds Leeds United Kingdom; 7 Leeds Institute for Data Analytics Leeds United Kingdom; 8 Leeds Institute of Cardiovascular and Metabolic Medicine University of Leeds Leeds United Kingdom; 9 Department of Cardiology Leeds Teaching Hospitals NHS Trust Leeds United Kingdom; 10 Keele Cardiovascular Group School of Medicine Keele University Keele United Kingdom; 11 Department of Health Sciences University of York York United Kingdom; 12 Leeds Institute of Health Sciences University of Leeds Leeds United Kingdom; 13 School of Medicine University of Leeds Leeds United Kingdom

**Keywords:** data, QualDash, audit, dashboards, support, quality

## Abstract

**Background:**

Dashboards can support data-driven quality improvements in health care. They visualize data in ways intended to ease cognitive load and support data comprehension, but how they are best integrated into working practices needs further investigation.

**Objective:**

This paper reports the findings of a realist evaluation of a web-based quality dashboard (QualDash) developed to support the use of national audit data in quality improvement.

**Methods:**

QualDash was co-designed with data users and installed in 8 clinical services (3 pediatric intensive care units and 5 cardiology services) across 5 health care organizations (sites A-E) in England between July and December 2019. Champions were identified to support adoption. Data to evaluate QualDash were collected between July 2019 and August 2021 and consisted of 148.5 hours of observations including hospital wards and clinical governance meetings, log files that captured the extent of use of QualDash over 12 months, and a questionnaire designed to assess the dashboard’s perceived usefulness and ease of use. Guided by the principles of realist evaluation, data were analyzed to understand how, why, and in what circumstances QualDash supported the use of national audit data in quality improvement.

**Results:**

The observations revealed that variation across sites in the amount and type of resources available to support data use, alongside staff interactions with QualDash, shaped its use and impact. Sites resourced with skilled audit support staff and established reporting systems (sites A and C) continued to use existing processes to report data. A number of constraints influenced use of QualDash in these sites including that some dashboard metrics were not configured in line with user expectations and staff were not fully aware how QualDash could be used to facilitate their work. In less well-resourced services, QualDash automated parts of their reporting process, streamlining the work of audit support staff (site B), and, in some cases, highlighted issues with data completeness that the service worked to address (site E). Questionnaire responses received from 23 participants indicated that QualDash was perceived as useful and easy to use despite its variable use in practice.

**Conclusions:**

Web-based dashboards have the potential to support data-driven improvement, providing access to visualizations that can help users address key questions about care quality. Findings from this study point to ways in which dashboard design might be improved to optimize use and impact in different contexts; this includes using data meaningful to stakeholders in the co-design process and actively engaging staff knowledgeable about current data use and routines in the scrutiny of the dashboard metrics and functions. In addition, consideration should be given to the processes of data collection and upload that underpin the quality of the data visualized and consequently its potential to stimulate quality improvement.

**International Registered Report Identifier (IRRID):**

RR2-10.1136/bmjopen-2019-033208

## Introduction

### Background

Health care organizations are complex systems with variations in patient care and outcomes observed nationally and internationally [1,2]. Audit and feedback may help reduce variations in care quality by comparing clinical performance against standards and benchmarks to stimulate data-driven improvement [3]. In the National Health Service (NHS) in England, national audit and feedback are part of a well-established quality improvement program that encompasses over 50 clinical specialties and patient groups [4,5]. Audit suppliers centrally collate and manage data from participating services and produce feedback, with national comparators, with the intention of stimulating quality improvement [6]. The mode and frequency of feedback varies between national audits but includes paper and electronic formats [7]. Feedback, however, is not used uniformly by participating services to stimulate quality improvement [4,6]. Reported constraints on the use of feedback include access to data, data timeliness and quality, metric relevance, and limited resources for data analysis and interpretation [7,8].

Dashboards offer the potential to overcome some of the constraints reported in the use of national audit data. They use visualization techniques intended to ease cognitive load and improve data comprehension [9,10]. In health care, a distinction is made between clinical dashboards that display performance at the level of individual clinicians or patients to inform direct patient care and quality dashboards that show performance at the level of a ward or organization to inform service improvement [11,12]. Despite the increasing use of dashboards in health care, including as part of the recent response to COVID-19 [13,14], evidence regarding how they become integrated into work processes to impact practice is limited [5]. The aim of this study, therefore, is to investigate how, why, and to what extent a novel quality dashboard (QualDash) supported the use of national audit data for quality improvement in an English hospital setting.

### Dashboard Development

QualDash is a customizable, web-based dashboard that was designed and evaluated using data from 2 national audits: The Myocardial Ischemia National Audit Project (MINAP) and the Pediatric Intensive Care Audit Network (PICANet). MINAP collects data spanning 130 data fields, contributed by all hospitals in England, Wales, and Northern Ireland that admit patients with acute coronary syndromes [15,16], whereas PICANet collects data from hospitals and services that transport critically ill children to pediatric intensive care units (PICUs) in England, Scotland, Wales, Ireland, and Northern Ireland [5,17]. These 2 audits vary in clinical specialty and metrics. For example, PICANet takes place in pediatric intensive care and includes metrics such as:

- Standardized mortality ratio: Ratio between the observed number of deaths and the number of deaths that would be expected, given the severity of patients’ illness at admission
- Unplanned extubations—accidental removal of breathing tubes
- Emergency readmissions within 48 hours of discharge

In comparison, MINAP audits services for heart attack and includes metrics such as:

- Call-to-balloon time: Time between ambulance call and primary percutaneous coronary intervention treatment: Target 90 minutes
- Door-to-angiography time: Time between arrival at hospital to diagnostic procedure: Target 72 hours
- Discharge on gold standard drugs: Proportion of patients who received all secondary prevention medication for which they were eligible

These audits were chosen for dashboard development to increase the generalizability of the findings beyond a single audit.

**Figure 1:**
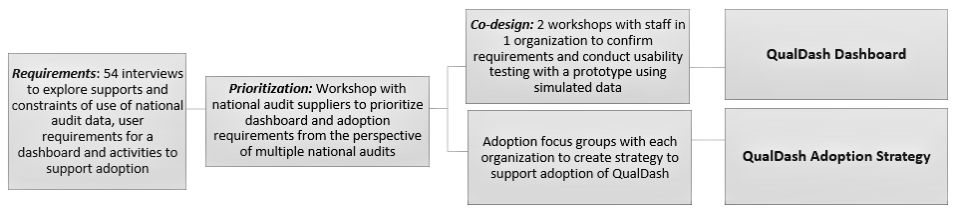
QualDash development work.

Dashboard development was conducted within 5 NHS acute health care organizations and included interviews with 54 staff members, a workshop with audit suppliers, and 2 co-design workshops with clinicians and managers from one organization [5,7,8,18]. Focus groups were held within each organization to identify strategies to support the uptake and adoption of QualDash. Figure 1 gives an overview of the development work.

**Figure 2:**
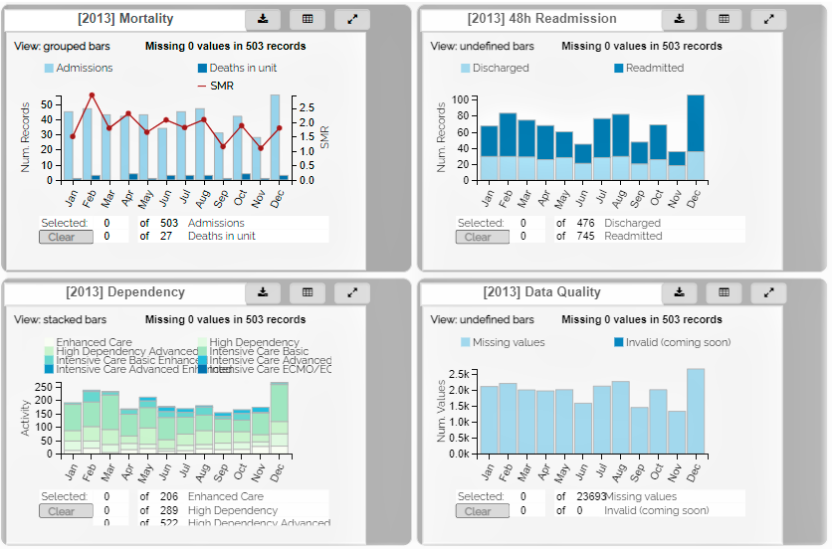
Four Pediatric Intensive Care Audit Network Qualcards—displays simulated data.

Development work provided insight into how national audit data were used—*user tasks*—and by whom in each organization, and what interrogative and reporting functions a quality dashboard should incorporate to facilitate data use. Requirements were documented in a software requirements specification [11] and were translated into a card metaphor, that is, metrics were configured as bar charts in moveable, customizable areas termed *Qualcards* using a metric specification structure in JavaScript Object Notation. Figure 2 shows an example of the QualDash prototype, displaying 4 Qualcards.

**Figure 3:**
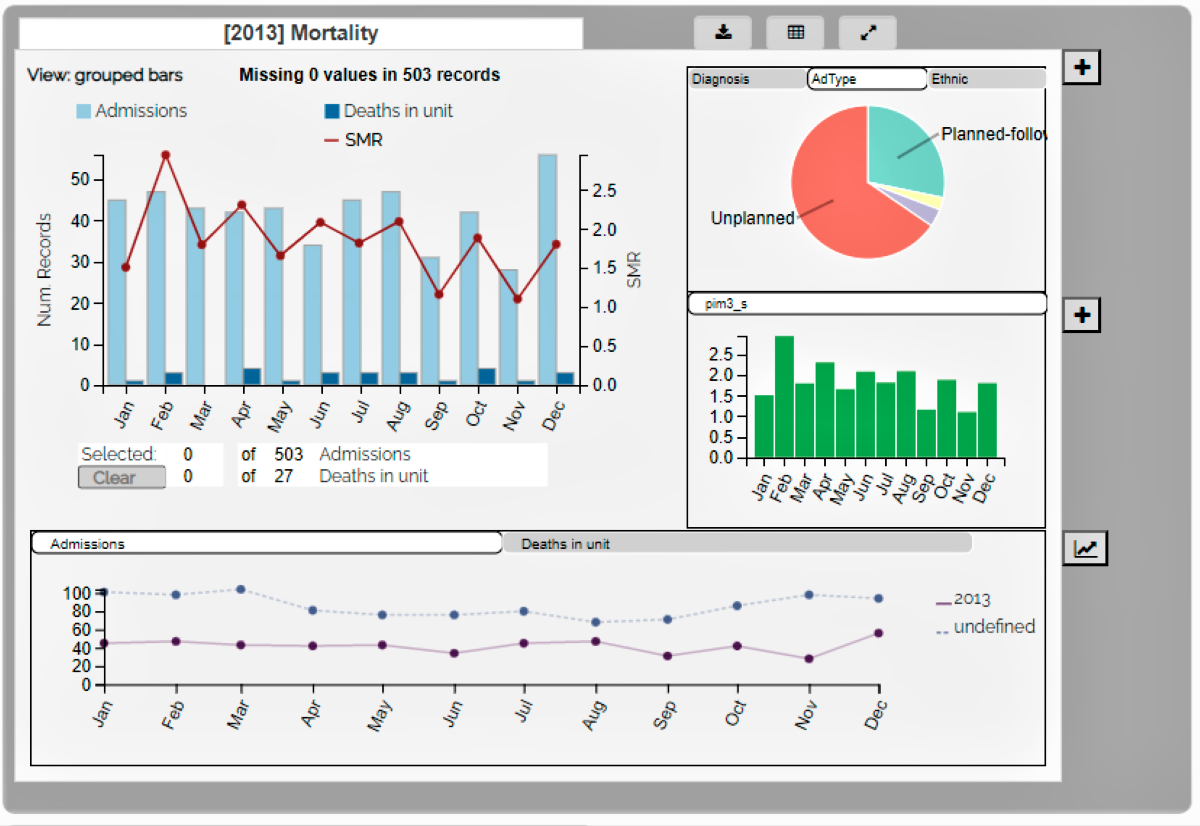
Expanded mortality Qualcard showing method of admission in the subview pie chart—displays simulated data.

Each Qualcard was designed to address a sequence of user tasks related to a single metric. On first opening QualDash, the user would see the main visualization for each metric displayed by month over a year; essentially, these could be used to address key care quality questions quickly and easily, for example, referring to the Mortality Qualcard in Figure 2, a user could address the question of how many deaths were reported in a unit last month compared with previous months? Qualcards were expandable (Figure 3) to display additional and historic measures to answer follow-up questions, for example, what was the method of admission for patients who died on a unit in a given month? Thus, QualDash was designed to facilitate care quality monitoring and data interrogation, where needed.

QualDash included functions to export visualizations and raw data and incorporated customization features, for example, users could select which variables were displayed in expanded Qualcard subviews, and dashboard authors (local staff with sufficient information technology [IT] experience) were able to configure service-specific variants of a metric, that is, add or adapt existing Qualcards. Usability evaluation of the prototype dashboard (Figures 2 and 3) was undertaken by staff from 2 organizations, using a think-aloud protocol and simulated data. Having completed a series of tasks, participants completed the System Usability Scale [19], with results suggesting a high level of usability.

Timeliness of data have also been reported to be important for data use in quality improvement. Managing the dashboard on a central server would have required requesting data from the national audit supplier and then waiting for those data to be sent to the research team before being uploaded to QualDash. Instead, to visualize data that were as timely as possible, QualDash was installed on site servers at each participating organization to enable staff to upload local data as needed. QualDash was accessible via Google Chrome on the service PCs. However, locating QualDash on local servers meant the dashboard displayed site data only; the metrics were relevant and used by services nationally but did not include national comparator data. Therefore, rather than comparing performance against peer organizations, sites compared service performance month-by-month and against national targets, for example, time to treatment targets.

## Methods

### Study Sample and Dashboard Installation

**Figure 4:**
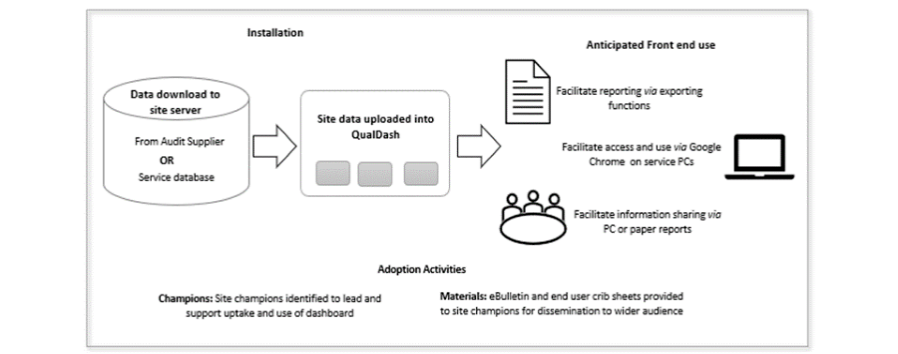
QualDash—data flow and anticipated front-end use.

QualDash was installed in 5 organizations including 3 large teaching hospital trusts that offer specialist services, including PICUs, and 2 smaller district general hospitals. Within these sites, QualDash was evaluated in services that participated in the 2 national audits of interest: PICANet and MINAP. District general hospitals do not provide the PICU service for which PICANet operates; therefore, the PICANet dashboard was available within PICUs in the 3 teaching hospital trusts and the MINAP dashboard was available in all 5 organizations. Figure 4 depicts how service-specific, real data were uploaded to QualDash on installation and anticipated front-end use.

To promote confidence in the accuracy of the data displayed, site data were validated by the research team on upload, after which site staff were able to upload data at their convenience. To support the adoption of QualDash and informed by the adoption focus groups, the research team identified *champions*, typically the study collaborators at each site, to lead, and support others in the use of the dashboard. Champions were provided with an eBulletin to advertise QualDash and crib sheets, with instructions on how to use dashboard functions, to disseminate as they saw appropriate within their organization. Furthermore, champions were encouraged to provide feedback about the dashboard that could be used to improve functionality where possible.

### Evaluation Framework

QualDash was conceptualized as a sociotechnical intervention [20-22]. Therefore, we sought to understand how interactions between the technological (the dashboard itself and existing information technology [IT] systems) and social (individuals and groups) system elements shaped dashboard use and impacts. For this purpose, we used realist evaluation, a theory-driven approach [23]. Realist evaluation is appropriate for evaluating sociotechnical interventions because it acknowledges that intervention impacts vary according to participants’ responses to the intervention, which are highly dependent on context. It provides a way to understand such variations using the concepts of mechanism, context, and outcome. Mechanisms refer to how recipients respond to, reason about, and interact with intervention resources, whereas context refers to factors that influence how mechanisms operate, including personal attitudes, beliefs, cultures, and resources [24]. Outcomes refer to the intended and unintended or unexpected impacts of interactions between mechanisms and contexts. Evaluation by this mode involves a cycle of constructing, testing, and refining realist theories configured as Context + Mechanism = Outcome, that is, hypotheses of how, why, and in what circumstances an intervention might work [23].

Multimedia Appendix 1 shows 2 Context + Mechanism = Outcome configurations (CMOcs) constructed using data collected as part of dashboard development [5,7,8], which will be used to illustrate the main study findings. In summary, we understood through development work that there was variable use of national audit data before QualDash, largely because of differences in the resources allocated to data collection and management across sites. However, clinicians used and acted on data if it helped them monitor whether their service was delivering safe and effective care, a mechanism we termed *professionalism*. We expected that QualDash, underpinned by this mechanism, would be used to facilitate data reporting (CMOc 1) or to integrate national audit data into routine reporting (CMOc 2) depending on existing use of data and resources, as illustrated in Multimedia Appendix 1.

### Extent of Use of QualDash

To assess the extent of use of QualDash across organizations and services, log files automatically recorded information about the use of QualDash at each site from the time of installation (June and July 2019) to July 2020. The information captured included the type of user (job title), data used (audit, year), time spent interacting with different Qualcards, and functionality used. Data from the log files were postprocessed in Microsoft Excel, removing entries generated by members of the research team undertaking on-site testing of the software. Using the timestamps for each login, the number of sessions per audit per month from installation to the end of July 2020 was determined for each site. Where a login occurred less than 20 minutes after the last timestamp and appeared to be the same user (based on the audit and year selected and the job title entered), this was treated as a continuation of the previous session.

### Observations of Practice

To understand how, why, and where QualDash was used, observations of practice were conducted at study sites between August 2019 and February 2020, in spaces where QualDash had the potential to be used in the ways expressed in the CMOcs, including:

- Hospital wards and units (eg, admission wards, catheterization laboratories, acute coronary care units, and PICUs)
- Clinical governance and directorate meetings
- Offices used by audit support and clinical staff for data management and administration activities
- Informal interviews relating to the activities observed

An observation schedule was developed to help guide data collection, including prompts to capture (1) service processes and routines for monitoring care quality, (2) types of data and technologies used and by whom, and (3) how, why, and the extent to which QualDash was integrated within these routines. Local collaborators within each service facilitated observations by introducing researchers to ward matrons and senior nurses. These staff provided permission for the observations to take place and notified their colleagues. Observations were conducted by 2 researchers who recorded the observations in handwritten notes. The observations were nonparticipatory, but researchers interacted with participants via informal interviews where an explanation of the activity observed was provided by the participant. The 2 researchers initially conducted observations together to develop a shared understanding of how to record fieldnotes, after which each researcher aimed to conduct at least one observation per service. Table 1 shows the total number of hours and types of observations conducted at each site.

**Table 1:** Hours and type of observation performed in study sites.

	Teaching Hospital Trust (MINAP^a^ and PICANet^b^)			District General Hospital (MINAP)		Total hours
	A	B	C	D^c^	E	
QualDash installation and customization meetings	9	8	16	7	6.5	46.5
Ward and “back office” observations, including data collection and validation	25	24.5	17	2	13.5	82
Meeting observations and informal interviews	7.5	3	4	3	2.5	20
Total hours of observation	41.5	35.5	37	12	22.5	148.5

^a^MINAP: Myocardial Ischemia National Audit Project.

^b^PICANet: Pediatric Intensive Care Audit Network.

^c^QualDash installed in December 2019 because of delays in site approval. This explains why the first use of QualDash at this site, discussed in results, begins in January 2021, much later than at other sites.

Observation notes were transcribed by researchers as soon as possible after the observation and analyzed following (and adapting) the 5 stages of framework analysis [25-28]. To familiarize themselves with the data (stage 1 of Framework Analysis), the transcripts were read independently by 3 researchers who then met to discuss their interpretations and construct themes to categorize the text (stage 2 of the Framework Analysis). The themes were uploaded into NVivo (QSR International; software for facilitating qualitative data analysis) and systematically applied to all transcripts (stage 3 of framework analysis) by the 2 researchers who conducted the observations, with themes being refined and added as necessary to capture the range of experiences. Instead of *charting* (stage 4 of Framework Analysis) where data are organized in charts of themes, NVivo was used to extract data categorized in each theme; these were then summarized in narrative accounts, for example, of the care quality monitoring processes observed, and the data and technologies used in these processes. To test CMOcs, data relevant to each CMOc (identified in the analysis work conducted) were summarized in a matrix developed in Word that displayed each CMOc construct by service. In this way, the CMOcs were refined by comparing observational data from each site against the hypotheses. Researchers used observational data to support the interpretation of the log file analysis by examining if it suggested an explanation for extent of use, for example, if QualDash was being used because it facilitated the work of audit support staff.

### Perceived Use and Usefulness

At the end of the evaluation period, a questionnaire based on the technology acceptance model (TAM) was administered [29]. The TAM consisted of 12 statements, 6 concerning usefulness, and 6 concerning ease of use. Respondents rated each statement on a scale of 1 to 5, with 1 indicating strong disagreement and 5 indicating strong agreement. In addition to the TAM items, the questionnaire included questions regarding how frequently respondents use national audit data, how frequently they used QualDash during the evaluation period, and how likely they would be to continue using QualDash after the evaluation period. A link to the questionnaire was emailed to 35 participants, who were known to have either used QualDash or seen it demonstrated at the beginning of August 2020. Two reminder emails were sent, and the survey was closed at the beginning of September 2020.

The questionnaire data were analyzed to assess the perceived usefulness of QualDash and its ease of use. Excel was used to produce summary statistics for each TAM statement, calculating the mean and range for all participants and for those who reported having used QualDash, broken down by audit and role. Guided by previous studies, ratings of 3 or higher were considered to indicate a positive response.

### Ethical Approval

Ethical approval for this study was provided by the University of Leeds School of Healthcare Research Ethics Committee (approval # HREC16–044). Approval was received from the Health Research Authority and each of the 5 sites.

## Results

### Extent of Use of QualDash

**Figure 5:**
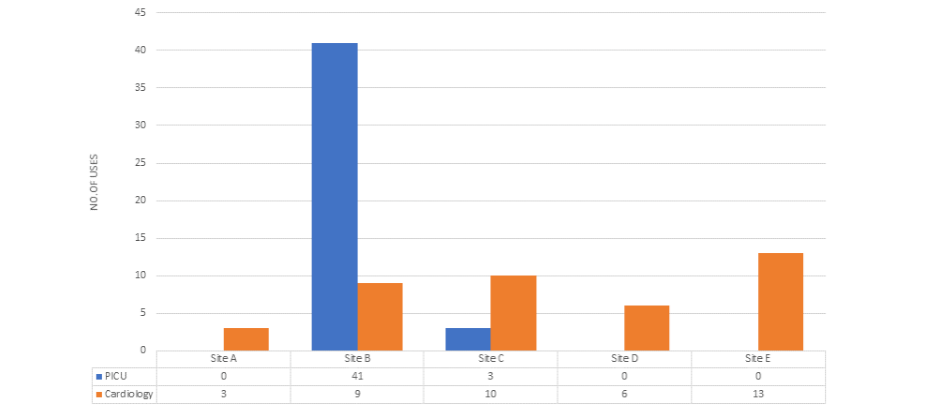
Extent of QualDash use by site and clinical service. PICU: pediatric intensive care unit.

**Figure 6:**
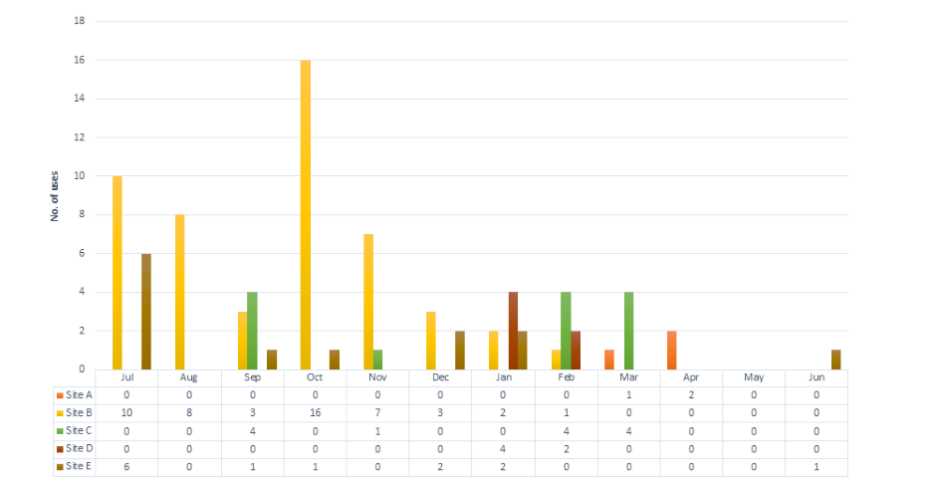
Uses of QualDash by clinical service and month.

Figure 5 shows the total number of uses of QualDash per clinical service and by site, and Figure 6 shows the number of uses by site over the evaluation period. The figures show variations in the extent of use across sites and clinical areas, for example, in site A, there was no use of QualDash within the PICU throughout the evaluation period (there could be no PICU use in sites D and E that did not have PICUs), whereas the greatest use was in the site B PICU and site E Cardiology.

Below, we use the observation data to understand the reasons behind these variations and to refine the CMOcs in Multimedia Appendix 1. We begin with a summary of the care quality monitoring routines observed, and then, to refine the CMOcs in Multimedia Appendix 1, explore how, why, and to what extent the dashboard impacted practice by facilitating reporting (CMOc 1) or integrating national audit data in routine practices (CMOc 2).

### Observations of Practice

#### Quality Monitoring Routines

The services observed included PICUs, where clinical staff cared for children, often on ventilators to assist their breathing, and cardiology wards for adults in admission for, and treatment or recovery from, acute coronary syndrome. Quality monitoring processes varied across services but included ward activities directed at the care of individual patients and directorate and clinical governance meetings directed at service-level monitoring of quality and safety.

Ward observations were conducted at or around the nurses’ stations. As might be expected, staff routines focused on the needs of individual patients, for example, ward staff shared information about patient care via handovers (summaries at shift changes), ward rounds, and clinical team *huddles* where staff met intermittently throughout the day to update colleagues about each patient’s condition and care plan. The use of technologies and data on all wards also focused on individual patient care. For example, electronic whiteboards and dry-erase whiteboards were used to visualize data in matrices, for example, patient by task or activity such as if they were awaiting a discharge letter. The use of patient case notes in paper form or electronic devices, such as PCs, laptops, and iPads positioned around the nurse’s station was also frequently observed. QualDash, however, was not observed to be used by staff on wards, despite opportunities via the electronic devices available to them.

**Figure 7:**
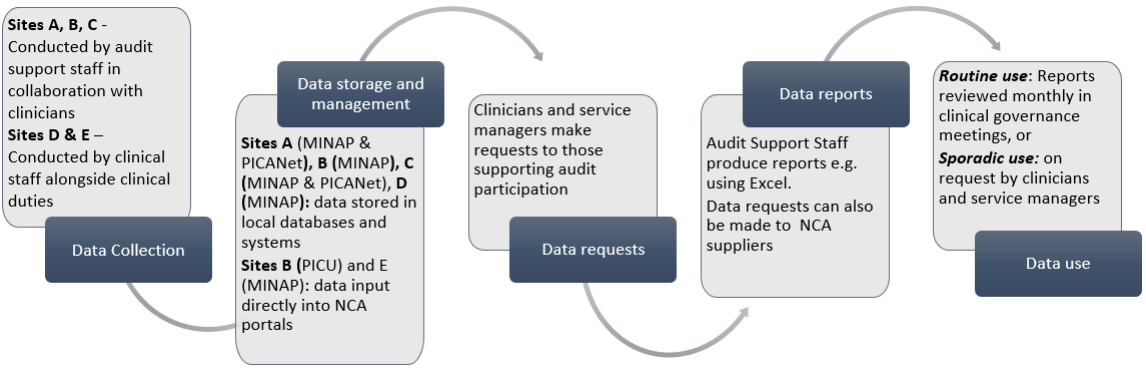
Audit data collection, management, and use. MINAP: Myocardial Ischemia National Audit Project; National Clinical Audit; PICANet: Pediatric Intensive Care Audit Network; PICU: pediatric intensive care unit.

The clinical governance (service) and directorate (specialty) meetings were observed monthly or quarterly. These meetings were attended by a range of staff, including nurses, physicians, junior doctors, and service managers. In comparison to the wards, observations revealed that national audit data were used in these meetings at certain sites, sometimes via QualDash. Figure 7 shows the flow of audit data from collection to practice, captured during observations.

Figure 7 is a simplified model of data use, showing how some services were resourced to use national audit data within their routine monitoring processes, whereas in others, use of national audit data was more sporadic*,* usually where clinicians were not supported by dedicated audit support staff. Next, we discuss the extent to which QualDash was integrated within work routines, and explore interactions that help explain the extent of use captured in the log files.

#### Facilitating Use of National Audit Data

The CMOcs hypothesize that, depending on the context, QualDash would be used either to facilitate the use of, or introduce, national audit data into service monitoring processes, such as the clinical governance meetings depicted in Figure 7. In practice, constraints were reported that limited the use of QualDash in these ways.

Sites A and C (PICU and Cardiology units) were resourced with dedicated audit support staff who produced performance reports when requested by clinicians and managers. Reports were produced using local databases and systems such as Microsoft Access or Microsoft Excel, where national audit data were stored before upload to audit suppliers. Observations revealed that, within these services, audit support staff continued to use existing systems post installation of QualDash. In site A Cardiology unit, for example, the nurse dedicated to audit participation did not have Google Chrome installed on their PC, despite repeated requests to their IT department, so they were unable to open QualDash to support reporting requests. Even in services where QualDash was accessible via Google Chrome, however, audit support staff continued to use existing systems, as this observation of an audit clerk updating monthly reports using their access database highlights:

We discuss a Qualcard that shows invasive ventilation and bed days. The Audit Clerk checks the figures in Qualcard against her visualizations for the same months, and they do not match. They explain that this mismatch might be because QualDash shows bed days by admission date rather than bed days across the months of admission. In one month, the Qualcard showed that there were more invasive ventilation days than there were bed days (which is impossible). Furthermore, the data in QualDash have not been updated since the installation (in July); therefore, the data in the access database is the only way to update the reports discussed today.

site A; PICU Observation; September 4, 2019

The use of simulated data in the co-design workshops meant that staff were unable to scrutinize the dashboard with data familiar and meaningful to them at that time. It was only post installation; therefore, when using QualDash to explore site specific data that it became apparent that some metrics were not configured as typically reported by the service. The issue described above was addressed by incorporating additional functionality within the dashboard, allowing Qualcards to be configured to present information in their respective months. The dashboard customization feature also supported metric reconfiguration. However, QualDash was not able to configure all of the metrics reported locally. For example, the number of accidental extubations (removal of a breathing tube) on PICUs per month could not be configured in QualDash because of the complex way in which data were captured as part of the audit. The measure was provided as the total number of incidents across the year in the subview pie chart but could not be used to facilitate monthly reporting. Similarly, in the site C Cardiology unit, a MINAP project assistant commented:

I would really love to be able to use it [QualDash], but it just does not show me, you know, everything I need, and I would like to be able to say to you, yeah, I use it all the time to add. But [...] it just does not do exactly what I need. But maybe if you spoke to someone like, the lead consultant [physician], and you showed him what was available, and you said, does this answer, you know, some of your queries? However, like the thing he asked me to do, to look at the patients that have failed [within specific time points].

site C; MINAP Project Assistant, Interview February 2020

The request to which the MINAP assistant refers was to see the data of patients treated between specific hours in one day. In the work undertaken to capture requirements for QualDash, tasks using such a timeframe were not identified; thus, this functionality was not included in QualDash.

In comparison to the dashboard itself, user knowledge of the audit data set and understanding of QualDash’s functionality also constrained use of the dashboard; an audit clerk in site C reported:

The Audit Clerk says that the service database contains more detail than PICANet, for example, when they produce reports for the business meeting, [...], they include information about what ward the patients have been referred from and where the patient was discharged. They also get “ad hoc” requests for data on a regular basis.

site C; Informal interview with PICU Audit Clerk; October 16, 2019

In fact, QualDash (and the PICANet data set from which it was derived) provided referral and discharge information in Qualcard subviews but this participant was unaware that the dashboard could support this data need. Even so, an additional influence on staff choice to use QualDash in these well-resourced services was that the data displayed were not as timely as those stored in local systems. This was due to challenges in establishing routines for uploading data into QualDash including having the necessary software installed on service PCs to complete the upload. Therefore, despite the manual work involved in report production, audit support staff in these services chose to use existing systems as they provided data that were timely and from which they had expertise that enabled them to configure metrics reported routinely and when requested by clinical team members.

In comparison to sites A and C, the audit clerk in site B PICU consulted hard copy and electronic data sources to submit quarterly reports to NHS England (a body of the Department of Health responsible for commissioning NHS services). Data sources included the ward diary, admissions books, and the patient administration system, and they also consulted with clinicians to retrieve data. QualDash automatically calculated measures, including patient bed days, 48-hour readmission, and mortality rates. Consequently, this audit clerk chose to use QualDash as it facilitated and streamlined their reporting, as hypothesized in the CMOc. Extent of use of QualDash in this site is reflected in Figure 5, and the impact captured in this email sent to the research team: “Just wanted to say QualDash has saved me hours of work with regard to data I submit to NHS England.”

An important observation was that, unlike other sites, this audit clerk developed a routine for uploading data into QualDash in a timely way. Therefore, where staff understood and experienced the benefits of using QualDash in their work routines, they supported the data upload process necessary to maintain dashboard use in practice.

#### Integrating National Audit Data Into Routine Monitoring Processes

Sites D and E were not resourced by staff dedicated to national audit data collection and management. These sites represented an opportunity for QualDash to be used to integrate national audit data within routine monitoring processes. QualDash was observed to be used within these sites, but not in the ways expressed in the CMOcs. For example, in site E, a cardiologist (and champion) was initially keen to use national audit data via QualDash. However, at the postinstallation site visit, the cardiologist noted:

The cardiologist says that they have not used QualDash since we first emailed the link to them in the installation email. When asked why, they explain that they have not yet integrated QualDash into their routines. They say that they are not accustomed to having access to MINAP data in this way. Therefore, they sometimes forget that it is there to look at and use QualDash. However, they say that there is no reason why they should not use it, and that one way in which they could try and integrate it into their practice is in their performance meetings where such data could be reviewed.

site E; informal interview; November 27, 2019

Despite being involved in the development and dissemination of QualDash as a champion, this physician and their service had managed without routine use of national audit data, and unfamiliarity with access to the data appeared to constrain their interactions with QualDash. The cardiologist added QualDash to the agenda for the next directorate meeting, which was observed as part of the data collection. During the meeting, the functions of QualDash were demonstrated to attendees, including physicians and nurses, and some tentative impacts were highlighted:

The cardiologist says that when QualDash was first installed, it enabled them to see where there was missing data and the audit team cleaned up the data in response. Therefore, QualDash has already helped the service to get their data a bit cleaner.

site E; Cardiology Directorate Meeting; December 11, 2019

The visualizations in QualDash enabled the audit team to identify missing data and work to address the gaps. However, the meeting chair queried the accuracy of the data displayed:

The meeting chair queries the displayed data. They say that all the graphs steadily decline and that “that cannot be accurate.” They ask [the Cardiologist who acted as QualDash Champion] why the graphs are like that, who responds that the audit team must be behind with the data collection. The Chair says that it is “amazing” to have access to real-time data, and that they should be able to make more use of MINAP data using QualDash.

site E; Cardiology Directorate Meeting; December 11, 2019

The meeting attendees responded positively to QualDash’s potential for supporting data use, but the data currently displayed could not be used optimally for quality improvement, because they were incomplete. This finding highlights the importance of the work underpinning front-end use of the dashboard, that is, data collection and upload, and the significance of the dedicated audit support staff in sites better resourced to use national audit data for quality improvement.

In site D, queries about the metric configurations discussed during a postinstallation meeting (QualDash was installed at this site in December 2019) raised concerns about the use and dissemination of the dashboard.

[Referring to a configuration of a complicated measure—patients with a certain diagnosis discharged on “gold standard” (up to five) drugs by month and for which the research team has received conflicting definitions] Cardiologists comment that they have come across this sort of thing before with new systems that are developed by people outside their unit: the data going in are correct, but it does not make sense, and that time is needed to check it to ensure that the data are interpreted correctly. They know what their own data should look like, though people outside the unit, including managers from their trust, do not always have that understanding and can misinterpret data that are not displayed correctly.

site D Observation; January 15, 2020

On the basis of previous experience with similar initiatives, the champions requested that all Qualcards undergo a *sense check* by staff familiar with site data. The expectation was that this sense check would be completed before dissemination activities took place, due to concerns about data interpretation by staff less familiar with the data. This finding also highlights the role of audit support staff, that their skills and experience in data collection, management, and report production provide confidence in the outputs they create and that site champions would need a similar level of trust in the dashboard to lead its use and dissemination to a wider audience.

### Perceived Usefulness and Ease of Use Questionnaire

**Figure 8:**
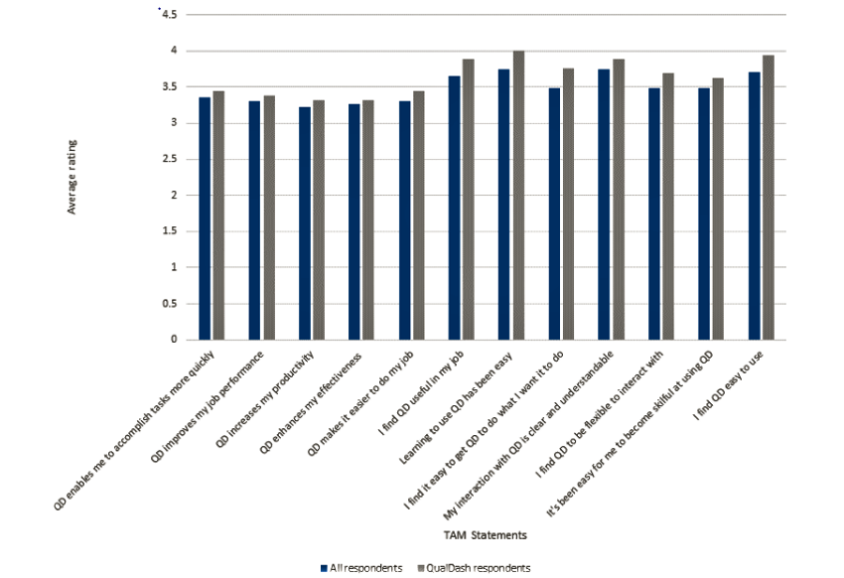
Rating of technology acceptance model statements. QD: QualDash; TAM: technology acceptance model.

In total, 23 responses were received from the adapted TAM questionnaire, 18 from participants who had experience using QualDash. Figure 8 shows the average ratings of each TAM statement.

Although the average rating for all statements was positive, ratings were higher among those who had used QualDash and higher for ease of use as opposed to usefulness. The questionnaire was anonymous, so we were unable to link responses to sites, but we were able to analyze them according to role, which revealed that audit support staff and doctors found QualDash more useful than nursing staff. On the basis of observations of ward practice, this finding is possibly a reflection of nurses’ more frequent engagement with data that informs direct patient care, as opposed to service-level data. Interestingly, however, observations suggested that senior nurses may find QualDash useful, as a pediatrician (and champion) commented:

I ask the physician if they see a role for QualDash in the PICU from what we have discussed. The physician says that QualDash is an “amalgamate of retrospective data” but it may help prospective planning, for example, highlighting high-risk patients. They say that they think senior nurses, bands 6 and 7, may be interested in the dashboard, and that QualDash icons on the nurse station PCs might be helpful in supporting their use of the dashboard.

Pediatrician; informal interview; site A; December 4, 2019

The pediatrician noted that QualDash displays aggregate, as opposed to patient-specific data, and that this may be useful for prospective planning on the wards. Therefore, senior nurses might utilize the dashboard if desktop icons facilitate access at the nurses’ station. Despite constraints on the use of QualDash during the evaluation period, its potential as a useful tool for a range of health care staff was recognized and ideas to support uptake in these ways suggested (desktop icons).

### Refinement of CMOcs

CMOc 1 in Multimedia Appendix 1 hypothesizes that QualDash would be used to facilitate the use of national audit data where data were used in care quality monitoring. We found that the use of data continued as usual during the evaluation period in services resourced with audit support staff and local systems that supported reporting needs. The reasons behind this choice encompassed dashboard functionality; it was not always possible to configure the Qualcards requested, some of which were used in routine reports—and contextual influences, for example, data were timelier in local systems where they were stored before uploading to QualDash and staff were not always aware of how QualDash could be used to facilitate their work. QualDash was used by an audit support clerk who recognized and experienced benefits (streamlined reporting process) from dashboard use in comparison to their existing system, and consequently established a routine for uploading timely data.

CMOc 2 in Multimedia Appendix 1 hypothesizes that QualDash would be used to integrate national audit data within routine monitoring processes where there were previously constraints accessing data. In one such site, we found that early interactions with QualDash led to additional requests to reconfigure Qualcards in line with user expectations. Work reconfiguring Qualcards was necessary to provide champions, across sites, with confidence to lead use of, and disseminate, QualDash within their service. In another service, we found that the dashboard visualizations highlighted missing data and that staff supporting audit participation could then work to address these issues. However, due to the missing data, QualDash could not be used optimally for quality improvement.

### QualDash Version 2 and the Impact of COVID-19

In response to user feedback, a new version of QualDash (Version 2) was developed that addressed issues with Qualcard configurations where possible, including invasive ventilation in PICANet and discharge of gold standard drugs in MINAP, as discussed above. The intention was to install Version 2 across all sites, continue dissemination activities, and capture further impacts via observations. However, in March 2020, the data collection and site visits were suspended due to COVID-19. The lockdown effectively ended face-to-face evaluation work, with data to refine CMOcs focused on learning from QualDash Version 1. Interestingly, the onset of COVID-19 has been accompanied by a rapid uptake in the use of digital technologies to deliver care in lockdown restrictions and to understand the impact of the virus on health care systems [30,31]. In this study, users in a site that previously made limited use of QualDash saw the potential benefits of the dashboard for responding to COVID-19 if it could be adapted to display data on a daily and weekly basis. Therefore, the computer scientist developing QualDash has worked to develop and install a new iteration of QualDash to support services in monitoring the impact of COVID-19.

## Discussion

### Principal Findings

This paper presents findings from a realist evaluation of a web-based, customizable, quality dashboard designed to support the use of national audit data in quality improvement. QualDash was co-designed with data users to address their needs, and champions were identified to support uptake and adoption. Even so, observations of practice revealed that QualDash had a variable impact across sites within the evaluation period. These findings are comparable with a recently updated review of dashboards that showed variation in impact, even within the same clinical area [32]. We used realist evaluation to understand the reasons behind the variation in impact.

Using CMOcs, we hypothesized that staff would integrate QualDash within their routines because it could facilitate monitoring and interrogation of metrics considered markers of safe and effective care—a mechanism we termed *professionalism*. This mechanism was identified in a context analysis of the use of national audit data and underpinned physicians’ use of data in quality improvement [7]. However, observations of practice revealed that when staff interacted with site-specific data via QualDash, they identified that some metrics were not configured in the format they expected. The dashboard’s customization feature enabled some metrics to be reconfigured to meet user needs, but it was not possible to configure all metrics requested or reported locally. Therefore, QualDash was not perceived as a tool that could facilitate data use as part of *professionalism* in some sites. Furthermore, not all services were resourced to upload data considered accurate or timely; attributes identified as influencing the use of national audit data and dashboards in health care more generally [6,7,14,33]. These factors constrained the use of QualDash in care quality monitoring, particularly where existing mature systems were in place to support data use.

A study of dashboard design in the context of lymphedema services reported that the complexity and accessibility of data to develop dashboards to support use of aggregate data was more challenging than the development of clinical, patient-level dashboards [33], and a review of data visualization dashboards has highlighted the need for user-centered design and interactions with a prototype to support dashboard development and implementation [34]. Our findings support this work and point to several ways in which the development of quality dashboards could be improved to support uptake and impact.

- First, the use of data familiar and meaningful to users in the co-design process would be beneficial. The use of site-specific data would encourage participants to scrutinize visualizations more thoroughly to confirm that they are configured as required for care quality monitoring before installation. Other studies have highlighted similar messages about how the interpretability of information needs greater consideration in design processes alongside usability and ease of use [35]. Our study highlighted that site champions need to have confidence that metric configurations are aligned with service expectations, to support interpretation by site staff, and to lead its use in quality improvement efforts.
- Second, engaging staff familiar with and knowledgeable about data use within the service, such as audit support staff and physicians, in scrutiny of dashboard metrics and functions, using real data, would provide an opportunity to (1) develop dashboard functionality in ways that can better support existing routines of data use and (2) raise awareness of the range of functionality offered by the dashboard among these key stakeholders, providing them with motivation to integrate the technology into their working practices on installation.
- The findings also emphasize the work surrounding and maintaining dashboard use. Alongside configuring metrics that reflect service needs, consideration needs to be given to the systems in place for collecting and uploading data in a timely and accurate way—attributes that also underpin confidence in the data and consequently the visualizations produced. These systems vary from service to service and the design of digital dashboards may need to be more ambitious, for example, incorporating solutions to automate data upload to support data timeliness and to ease the workload of those supporting audit participation where necessary. This may be particularly pertinent as health care providers around the world seek to move to paperless systems.

### Strengths and Weaknesses

A challenge when evaluating digital technologies is that development is *a continuous cycle* in which users adapt to the technology and adapt the technology over time [36]. Installation of a new iteration of QualDash (Version 2) was disrupted by COVID-19; therefore, it was not possible to capture the impact of this improved version on end use or impact. However, our in-depth data collection across multiple sites and clinical areas enabled us to explore how interactions between the social and technical elements of the system shaped dashboard impact. We were able to explore the reasons behind participants’ choice to use or not use QualDash, and the circumstances that influenced those choices, which has generated knowledge that can support the design process for quality dashboards outside the context of this study and strategies to support uptake and adoption.

### Conclusions

Web-based, customizable dashboards have the potential to support the use of national audit data in quality improvement, reducing variation in data use across services nationally, and, as COVID-19 has demonstrated, in response to specific events or crises. To optimize the dashboard impact in different settings, co-design would benefit from use of *real* site data, so stakeholders can scrutinize metrics and functions with data that are familiar and meaningful to them. In this way, dashboard development would more closely align with the needs and working practices of key data users within the service. Furthermore, data flow, from collection to upload, needs consideration in the design process, to provide insight into interdependent issues, such as data timeliness and quality, that are likely to impact dashboard use in quality improvement.

## Appendix

Multimedia Appendix 1 Context + Mechanism = Outcome configurations.

## Abbreviations

CMOc: Context + Mechanism = Outcome configuration
IT: information technology
MINAP: Myocardial Ischemia National Audit Project
NHS: National Health Service
PICANet: Pediatric Intensive Care Audit Network
PICU: pediatric intensive care unit
TAM: technology acceptance model
