# Supplementary material for: Analysis of a Web-Based Dashboard to Support the Use of National Audit Data in Quality Improvement: Realist Evaluation
Source: J Med Internet Res. 2021 Nov 23;23(11):e28854. doi: 10.2196/28854 (PMC8663683; doi:10.2196/28854)
Supplement: Multimedia Appendix 1 [file jmir_v23i11e28854_app1.docx]

Table 1 CMO configurations tested in stage 3 and 4.

| Context | | Mechanism | | Outcomes | |
| --- | --- | --- | --- | --- | --- |
| Circumstances | For whom | Intervention | Response | Local | National |
| Providers invest significant resources participating in national audits, but there is variable use in the outputs.  Providers operate in a context of financial incentives and constraints.  Clinical services / units have responsibility for care quality monitoring.  Adoption activities conducted to support uptake and adoption of QualDash. | **1**. Clinical services resourced to use national audit data routinely *via* audit support staff and local systems/ databases. | QualDash provides web-based access to key performance measures used in quality monitoring | Audit support staff use QualDash because it facilitates production of reports requested by clinical staff to monitor whether care is safe and effective. | QualDash streamlines report production for audit support staff, saving their time. | Reduced variation in use of national audit data, providing more opportunity to stimulate Quality Improvement. |
|  | **2**. Clinical services not resourced to use national audit data routinely | QualDash provides web-based access to key performance measures used in quality monitoring | QualDash Champions use QualDash in appropriate settings because it enables them and the service to easily access and monitor if care is safe and effective***.*** | National audit data integrated into routine monitoring process. |  |
